# Reappraisal of Risk Factors for T-Cell-Mediated Kidney Rejection

**DOI:** 10.1016/j.ekir.2026.106537

**Published:** 2026-04-13

**Authors:** Michiel G.H. Betjes, Judith A. Kal-van Gestel, Nicolle H.R. Litjens, Marcia M.L. Kho, Jacqueline van de Wetering, Marc H. Hemmelder, Annelies E. de Weerd

**Affiliations:** 1 Division of Nephrology and Transplantation, Department of Internal Medicine, Erasmus Medical Center Transplant Institute, University Medical Center, Rotterdam, The Netherlands

**Keywords:** donor-specific antibodies, graft survival, kidney, rejection, T-cell-mediated rejection, transplantation

## Abstract

**Background:**

The risk factors for and the impact of T-cell-mediated rejection (TCMR) in the long term after kidney transplantation in recipients receiving anti-cluster of differentiation (CD)25 induction, followed by tacrolimus or mycophenolate mofetil (MMF) or prednisone maintenance is unknown.

**Methods:**

A single center cohort of 2024 kidney transplant recipients between 2010 and 2020 with follow-up until June 2025 was analyzed retrospectively for frequency and type of TCMR, impact on kidney function, and graft loss. Potential risk factors for TCMR were evaluated.

**Results:**

The cumulative incidence of acute TCMR in the group without pretransplant donor-specific anti-human leukocyte antigen (HLA) antibodies (preDSA) (*n* = 1733, 86% of cohort) was 15% (4% borderline TCMR [bTCMR], 2% Banff TCMR grade 1, 7% Banff TCMR grade 2 and 3, and 2% mixed type rejection) after a median follow-up of 7 years. TCMR was associated with a lower estimated glomerular filtration rate (eGFR) at 1 year (43 ml/min per 1.73 m^2^ vs. 52 ml/min per 1.73 m^2^, *P* < 0.001), and TCMR-related graft loss at 10-year follow-up was 3%. Increasing incidence of TCMR was associated with younger age recipients, deceased donor (DD) kidney, and higher number of HLA mismatches. The presence of preDSA (*n* = 291, 14% of cohort) substantially increased the incidence of all types of TCMR (28% at 1 year for preDSA vs. 13% for no preDSA, *P* < 0.001) and TCMR-related graft loss (7% vs. 0.8% at 1 year, *P* < 0.001). This effect was mainly driven by the subgroup of recipients with preDSA against HLA class II.

**Conclusion:**

An immune suppressive regime of anti-CD25 induction and tacrolimus or MMF or prednisone maintenance is associated with a low risk of TCMR-related graft loss except for recipients with preDSA against HLA class II.

## Graphical abstract

**Figure:**
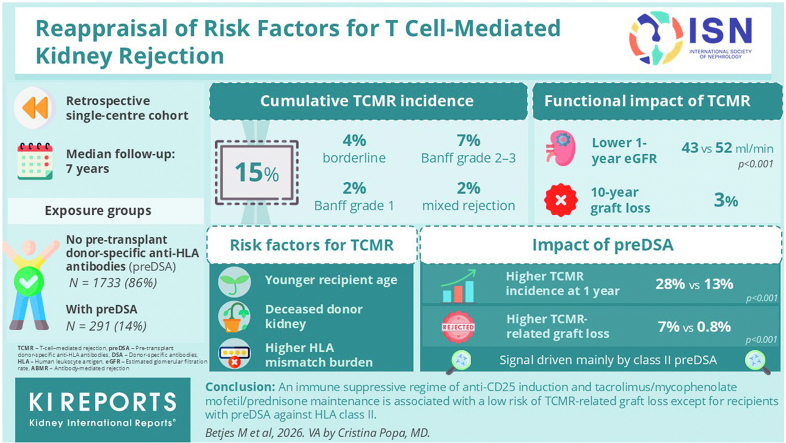

After the introduction of tacrolimus, most centers have replaced ciclosporin by tacrolimus as it is associated with less risk for acute TCMR.^1^^,^^2^ The landmark Efficacy Limiting Toxicity Elimination (ELITE) trial showed that an immune suppressive regimen of anti-CD25 induction with tacrolimus, MMF, and prednisone is associated with an acceptable risk of TCMR and good graft function and a low risk of opportunistic infections.^3^ Perceived high risk recipients, such as donation after circulatory death donor kidneys and a panel reactive antibody (PRA) > 20% were excluded in this trial with a follow-up of 12 months. Nevertheless, most transplantation centers in the USA and Europe prefer T-cell depleting induction, even in the growing population of older adult recipients.^4^, ^5^, ^6^ This may be because of the lack of long-term follow-up data on efficacy and adverse effects of the ELITE tacrolimus-based scheme without selection of recipients for T cell depletion or basiliximab.

The detection of DSA using the sensitive single antigen bead Luminex assay enables to identify recipients with preDSA with or without the presence of cytotoxic DSA. This has led to a major shift in research from TCMR to antibody-mediated rejection (ABMR), as the latter is highly associated with the presence or development of DSA.^7^ In addition, large patient cohorts have shown that preDSA are associated with a significant decreased graft survival via the development of chronic-active ABMR over time.^8^, ^9^, ^10^, ^11^ One can hypothesize that HLA sensitization to donor HLA as shown by the presence of DSA may also identify recipients at increased risk for TCMR. This may occur via the pathway of direct alloreactivity, that is, the recipient T cells having been primed with donor HLA molecules, or via the indirect pathway, as 2 studies showed that predicted indirectly recognizable HLA epitopes scores (as a measure for HLA epitopes which can be presented indirectly to recipient T cells) are associated with vascular rejection.^12^^,^^13^ However, the association between preDSA and incidence of TCMR has shown contradictory results.^14^, ^15^, ^16^, ^17^

At our center, the ELITE trial immunosuppressive regime has been adopted from 2009 for all type of donor kidneys and for all recipients receiving an ABO compatible kidney transplant with a complement-dependent cytotoxicity-negative crossmatch. We investigated the cumulative risk for TCMR and associated short- and long-term graft loss and reevaluated risk factors for TCMR taking preDSA into account, in a cohort from 2010 to 2020 with granular data.

## Methods

This study included all 2124 consecutive kidney transplantations performed between January 2010 and December 2020 at the Erasmus Medical Center in the Netherlands. The last follow-up date for data analysis was June 2025. Recipients were seen at least once a year at the outpatient clinic, and clinical data were registered in a national database (Netherlands Organ Transplant Registry) complemented with data from the local registry. The final database for analysis was > 98% complete for all variables. All transplantations across the ABO blood group barrier (*n* = 88) or a positive complement-dependent cytotoxicity crossmatch at time of transplantation (*n* = 12) were excluded from analysis. The clinical characteristics of the included 2024 patients are shown in Table 1.

**Table 1:** Kidney transplant recipients and donor kidney clinical characteristics (*N* = 2024)

Mean age recipient, yrs (SD)	55.7 (14.1)
Mean age donor, yrs (SD)	53.5 (14.5)
Recipient male/female ratio	62%/38%
Follow-up, yrs, median (IQR)	7.0 (4.5–8.6)
Deceased/living donor kidney	42%/58%
-DBD type	17%
-DCD type	23%
-Delayed graft function in DBD/DCD	30%/57%
-Never functioning graft^a^	2%
Cold ischemia time for DD donor kidneys	11.0 ± 3.9 h
Re-transplantation	16%
PRA^b^ > 5%	9.5%
HLA mismatches (median)	
Class I	2
Class II	1
Class I and II	3
preDSA (% of all recipients)	14
Anti-HLA class I/II/I+II	7%/2%/5%
% within each donor group: LDK/DBD/DCD	15/16/11
Induction therapy	96%
- Anti-IL-2 receptor antibody	94%
- T-cell depleting antibody	2%
Maintenance immune suppression	
- Steroids	100%
- tacrolimus/ciclosporin	99%/1%
- MMF/azathioprine	99%/1%
- Other	< 1%

DBD, deceased by brain death; DCD, deceased by circulatory death; DD, deceased donor; DSA, donor-specific anti-HLA antibodies; HLA, human leukocyte antigen; IQR, interquartile range; LDK, living donor kidney; MMF, mycophenolate mofetil; PRA, panel reactive antibodies.

a The category “never functioning graft” includes all kidney transplants that have never functioned sufficiently to allow stopping dialysis.

b PRA above 5% indicates the presence of serum cytotoxic anti-HLA antibodies.

The standard immune suppressive maintenance protocol consisted of tacrolimus (aiming for predose concentrations of 10–15 ng/ml in weeks 1–2, 8–12 ng/ml in weeks 3–4, and 5–10 ng/ml, thereafter) combined with MMF (starting dose of 1 g twice a day, aiming for predose concentrations of 1.5–3.0 mg/l) and glucocorticoids. All recipients received 50 mg of prednisolone twice a day i.v. on days 0 to 3. Thereafter, 20 mg of oral prednisolone was started and subsequently tapered to 5 mg at month 3 and thereafter stopped within 3 months.

The clinical and research activities being reported are consistent with the principles of the Declaration of Istanbul as outlined in the “Declaration of Istanbul on Organ Trafficking and Transplant Tourism” and in accordance with the Declaration of Helsinki. Patients gave written informed consent for participating in the Netherlands Organ Transplant Registry database. Approval for assessing additional clinical information was obtained by the institutional review board of the Erasmus Medical Center (MEC-2021-0357 and MEC-2024-0193). Study design and analysis were done in accordance with the STROBE statement.

All renal biopsies were for cause and were performed in case of progressive loss of graft function. The initial biopsy reviews were rescored following the 2018 Banff Reference Guide^18^ with bTCMR defined by the 2005 criteria. T-cell-mediated rejection was treated with methylprednisolone 1000 mg i.v. for 3 days in case of bTCMR or Banff TCMR grade 1 (tubulo-interstitial rejection). Banff TCMR grade 2 and 3 (vascular rejection) was treated with T-cell depletion therapy using rabbit antithymocyte Ig between 2010 and 2015 or alemtuzumab from 2015 to 2020. Mixed rejection was treated with methylprednisolone 1000 mg i.v. for 3 days and Igs (1-2 g/kg i.v.) and plasmapheresis in case of donor-specific antibodies.

### Outcomes and Variables

For data analysis on TCMR incidence and related graft loss the first-time diagnosis of TCMR was used. For classifying the cause of graft loss, the histology of the kidney biopsy was further categorized as previously published as follows^19^: rejection, recurrence of primary kidney disease, diagnosis of *de novo* kidney disease, and interstitial fibrosis with tubular atrophy. In case of graft failure, the diagnosis of for-cause kidney biopsies was used to categorize the type of graft failure if no other clinical event could explain the loss of kidney function.

The other graft loss categories were a clinical event leading to irreversible graft failure (e.g., circulatory shock, pyelonephritis, or graft thrombosis) and “unknown” if a clinical diagnosis for allograft failure could not be established, and no biopsy was performed (1% of all cases of graft loss other than death). Primary nonfunction is the category of grafts that never functioned after transplantation with no other diagnosis than acute tubular necrosis as shown by kidney biopsy. DSA were measured in this study as previously reported.^16^ The percentage of PRA at the time of transplantation, as determined by complement-dependent cytotoxicity assay, was considered positive when > 5%.

The relative contribution of TCMR-related graft loss was assessed at 1 year after transplantation and for the group of recipients with a follow-up of at least 10 years and no preDSA (Table 2). The latter group was selected to account for death with a functioning graft as a competitive risk factor within different recipient age strata.

**Table 2:** Kidney transplant recipients without preDSA with at least 10-year follow-up (*n* = 1086) and donor kidney clinical characteristics

Mean age recipient, yrs (SD)	57.7 (13.6)
Mean age donor, yrs (SD)	54.2 (14.6)
Recipient male/female ratio	67%/33%
Deceased/living donor kidney	42%/58%
-DBD type	17%
-DCD type	25%
-Delayed graft function in DBD/DCD	30%/57%
Never functioning graft^a^	1.4%
Pre-emptive transplantation	32%
Cold ischemia time for DD donors	12.8 ± 4.3 h
Re-transplantation	12%
HLA mismatches (median)	
Class I	2
Class II	1
Class I and II	3
PRA^b^ >5% Median % of positive PRA (min-max)	6.2% 25% (6–96)
Induction therapy	97%
- Anti-IL-2 receptor antibody	95%
- T-cell depleting antibody	2%
Initial maintenance immune suppression	
- Steroids	100%
- tacrolimus/ciclosporin	99%/1%
- MMF/azathioprine	99%/1%
- other	< 1%

DBD, deceased by brain death; DCD, deceased by circulatory death; DSA, donor-specific anti-HLA antibodies; HLA, human leukocyte antigen; MMF, mycophenolate mofetil; PRA, panel reactive antibodies.

a The category “never functioning graft” includes all kidney transplants that have never functioned sufficiently to allow stopping dialysis.

b PRA above 5% indicates the presence of serum cytotoxic anti-HLA antibodies.

Cause of death was categorized as infection-related, cardiovascular, malignancy, other, and unknown.

### Statistical Analysis

Differences in patient, donor, and transplant characteristics were assessed by the Fisher’s exact test for categorical variables and Mann-Whitney U test for continuous variables. All *P*-values were 2-tailed. The cumulative incidence of TCMR and death-censored graft loss was assessed by Kaplan-Meier survival analysis with log-rank statistics for difference between strata. Univariate Cox proportional hazards analysis was used to identify clinical and demographic variables associated with rejection and graft survival. Variables considered for analysis were as follows: donor age, age of recipient, retransplantation, pre-emptive transplantation, positive PRA, preDSA, number of HLA mismatches on A, B, and DR, type of kidney donor (living donor [LD], donation after circulatory death, and donation after brain death), male or female, and cold ischemia time. Variables with a *P*-value of < 0.1 in a univariate analysis were subsequently used in the multivariate Cox proportional hazard analysis with stepwise forward regression to calculate adjusted hazard ratios (HRs) for the outcome (e.g., TCMR or graft failure). Interaction terms that met statistical significance (*P* < 0.05) were included in the multivariate model. All adjusted HRs (aHRs) were calculated with the multivariate Cox proportional hazard analysis. Statistical analysis was performed with SPSS Statistics (version 21, IBM Corp, Armonk, New York). Statistical significance was met if the *P*-value was < 0.05.

## Results

### Clinical Characteristics of Recipients and Kidney Donors

The clinical characteristics of the included 2024 recipients, of whom 14% had preDSA are shown in Table 1. The median follow-up after kidney transplantation was 7 years. Thirty-eight recipients (2%) were lost to follow-up at a median time of 2 years after transplantation. In Table 2, the data are given for recipients without preDSA and a follow-up of at least 10 years.

### Risk Factors for TCMR

The cumulative incidence of TCMR for the entire cohort was 20%; 4% bTCMR, 4% Banff TCMR grade 1, 8% Banff TCMR grade 2 and 3, 4% mixed rejections, and 0.3% chronic active-TCMR.

Multivariate logistic regression showed that increasing recipient age (per year: aHR 0.98, 95% confidence interval [CI]: 0.98–0.99), donor age (per year: aHR: 1.01, 95% CI: 1.00–1.02), donor type (DD vs. LD kidney: aHR: 1.38, 95% CI: 1.12–1.70), number of HLA mismatches (aHR: 1.09, 95% CI: 1.02–1.16), and the presence of preDSA (aHR: 2.37, 95% CI: 1.88–2.99 were significantly associated with the risk of TCMR (Supplementary Table S1). These associations were observed for every type of TCMR (data not shown). The majority of TMCR occurred within the first year of transplantation with a significant higher incidence of TMCR within the preDSA group compared with the no preDSA group (13.3% vs. 27.8% for TCMR [*P* < 0.001], Table 3). A sensitivity analysis was performed by excluding all TCMR episodes beyond the first year, and the results showed similar aHR for the risk factors identified (data not shown).

**Table 3:** Incidence of TMCR within first year after kidney transplantation and TCMR-related graft dissected for the presence or absence of pretransplant donor-specific anti-HLA antibodies

Type of TCMR	Total cohort *N* = 2024		preDSAneg *n* = 1733		preDSApos *n* = 291		*P*-value preDSA vs. no preDSA
	Number of rejections^a^	TMCR-related graft loss	Number of rejections	TMCR-related graft loss	Number of rejections	TMCR-related graft loss	
TCMR							<0.001
Incidence first year	311	33 (10.6%)^b^	230	14 (6.1%)^b^	81	21 (25.9%)^b^	<0.001
% TMCR-related graft loss of all	15.4%^c^	1.6%^d^	13.3%	0.8%	27.8%	7.2%	<.001
bTCMR	67 (78.8%)^a^	2 (2.9%)^b^	54 (79%)^a^	0 (0%)^b^	13 (76.5%)^a^	2 (15.4%)^b^	0.03
Incidence first year	3.3%		3.1%		4.4%		0.28
TCMR 1	60 (73.25%)^a^	3 (5.0%)^b^	41 (69%)^a^	1 (2.4%)^b^	19 (82.6%)^a^	4 (21.0%)^b^	0.03
Incidence first year	2.9%		2.4%		6.5%		<0.001
TCMR 2–3	132 (86.3%)^a^	14 (10.6%)^b^	105 (87.5%)^a^	10 (9.5%)^b^	27 (81.8%)^a^	4 (14.8%)^b^	0.48
Incidence first year	6.5%		6.0%		9.2%		0.05
Mixed type	50 (63.0%)^a^	14 (28%)^b^	28 (73.7%)^a^	3 (10.7%)^b^	22 (53.0%)^a^	11 (50%)^b^	<0.001
Incidence first year	2.5%		1.6%		7.6%		<0.001
ca-TCMR	2 (25%)^a^	0 (0%)^b^	2 (25%)^a^		0 (0%)^a^	0 (0%)^b^	1.0
Incidence first year	0.1%		0.1%		0%	0%	

ABMR, antibody mediated rejection; bTCMR, borderline TCMR; ca-TCMR, chronic active TCMR; DSA, donor-specific anti-human leukocyte antigen antibodies; TCMR, T-cell mediated rejection.

Mixed-type indicates fulfilling criteria for both TCMR and ABMR.

a Between parenthesis the % of total number of rejections during follow-up.

b Between parenthesis the % of number of rejections within the first year leading to graft loss.

c %TCMR-related graft loss of all means; % of all graft loss within the first year which can be attributed to TMCR (in this case 15.4%).

d The 1.6% is the % of TCMR related graft in the first year as a % of all recipients (33 out of 2024).

**Figure 1:**
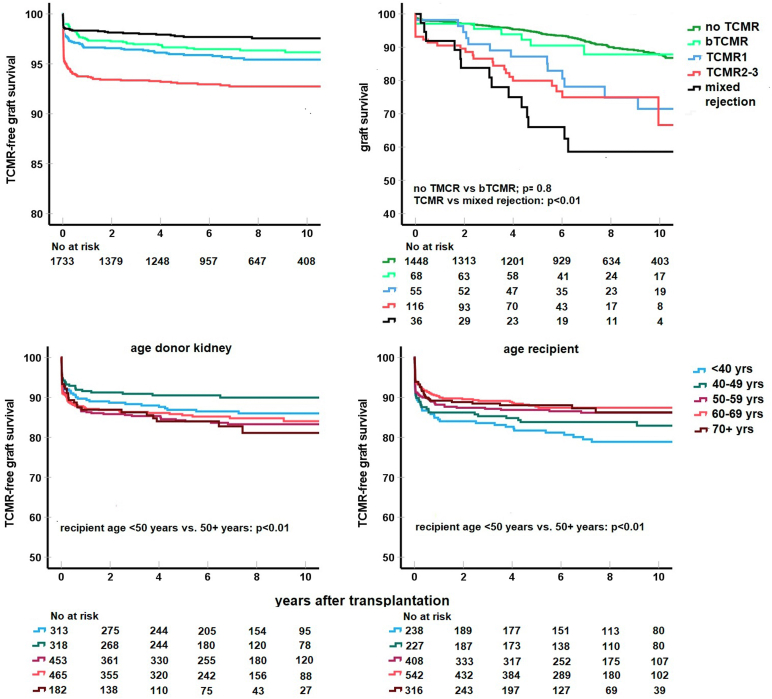
T-cell-mediated rejection (TCMR) free survival for different subtypes of TCMR (borderline TCMR [bTCMR], type 1, and type 2–3 and mixed rejection) in the recipients without preDSA (*n* = 1733) is shown in upper left panel. Death-censored graft survival within the different groups with TCMR with log-rank test for difference with the group without TCMR during follow-up (upper right panel). TCMR-free survival for different donor (left bottom panel) and recipient age groups (right bottom panel) with log-rank statistics for the difference between younger or older than 50 years. DSA, donor-specific anti-human leukocyte antigen antibodies.

In the group of recipients without preDSA (*n* = 1733, 86% of cohort), the cumulative incidence of acute TCMR was 15% (4% bTCMR, 2% Banff TCMR grade 1, 7% Banff TCMR grade 2 and 3, and 2% mixed type rejection) after a median follow-up of 7 years (Figure 1). Multivariate logistic regression showed that increasing recipient age (per year; aHR: 0.99; 95% CI: 0.98–0.99; *P* = 0.004), the total number of mismatches (per mismatch; aHR: 1.24; 95% CI: 1.04–1.21; *P* = 0.003), and type of donor organ (DD vs. LD kidney; aHR: 1.39; 95% CI: 1.09–1.77; *P* = 0.007) were significantly associated with the incidence of TCMR (Supplementary Table S2).

**Figure 2:**
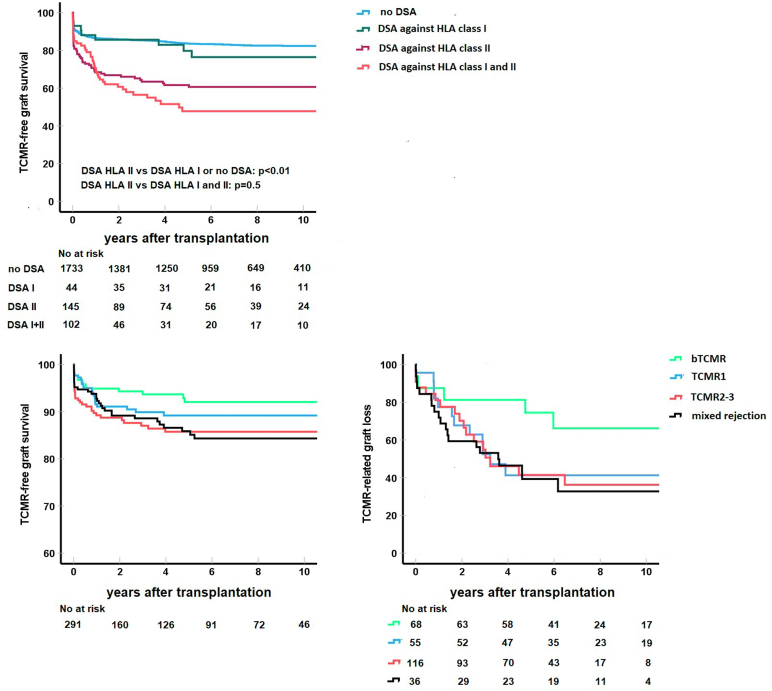
T-cell mediated rejection (TCMR) free survival in groups without and with different types of donor-specific anti-HLA antibodies (DSA) before transplantation (no or preDSA, upper panel) with log-rank test for comparison of anticlass II DSA versus no anticlass II DSA. The lower panels show incidence of TCMR (left) and TCMR-related graft loss in for different subtypes of TCMR (borderline TCMR [bTCMR], Banff TCMR grade 1, 2, and 3, mixed rejection) for the preDSA group. Recipient numbers at risk is shown below the upper graph. HLA, human leukocyte antigen.

In recipients with preDSA, increasing age of the donor (per year: aHR: 1.02; 95% CI: 1.01–1.04; *P* = 0.006) and the recipient (per year: aHR: 0.98; 95% CI: 0.97–0.99), *P* = 0.03) were associated with more and less TCMR, respectively. Only preDSA against HLA class II were significantly associated with an increased risk of TCMR (aHR: 2.13, 95% CI: 1.07–4.24, Kaplan-Meier curves in Figure 2).

In conclusion, in particular the presence of preDSA against HLA class II is associated with a substantial increase of the cumulative incidence of TCMR. Sensitization for allogeneic HLA *per se* (e.g., a positive PRA without DSA or a previous kidney transplantation) was not associated with the cumulative incidence of TCMR.

### Kidney Graft Loss Related to TCMR at 1 Year and 10 Years After Transplantation

Graft loss censored for death in the no preDSA group was not different in the bTCMR group compared with the group without TCMR, whereas it was increased in the other TCMR groups, specifically in the subgroup of mixed rejection (Figure 1). TCMR in the group with preDSA lead to increased graft loss compared with the group without preDSA (Figure 2, Table 3).

**Figure 3:**
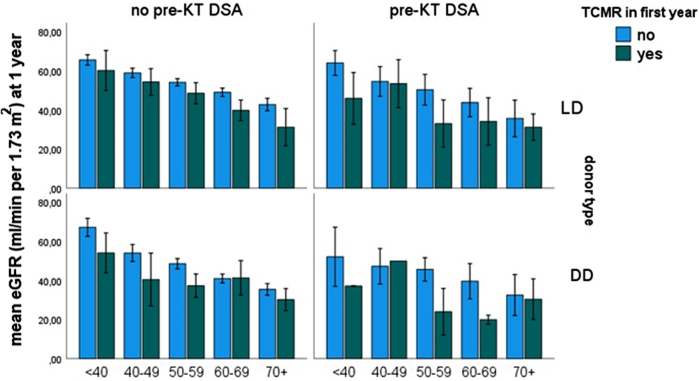
Estimated glomerular filtration rate (eGFR) in ml/min per 1.73 m^2^ at 1 year after transplantation or recipients with or without a T-cell-mediated rejection (TCMR) in the first year. Data are shown as means with SEM for different strata of donor kidney age categories, type of donor kidney (deceased donor [DD] and living donor [LD] kidney) and preDSA or not. DSA, donor-specific anti-human leukocyte antigen antibodies; KT, kidney transplantation.

TCMR-related graft loss at 1 year was higher in the preDSA group for each subtype of TCMR (Table 2). In the group without preDSA, 6% of all TCMR episodes led to graft loss which translated in 0.8% TCMR-related graft loss at 1 year in this group. In the preDSA group, this was 23% and 7%, respectively (*P* < 0.001). The impact of TCMR within the first post-transplant year on eGFR at 1 year was on average 8 ml/min per 1.73 m^2^ (no TCMR: 52 ml/min per 1.73 m^2^, bTCMR: 43 ml/min per 1.73 m^2^, TCMR1: 46 ml/min per 1.73 m^2^, TCMR2–3: 44 ml/min per 1.73 m^2^, mixed rejection: 42 ml/min per 1.73 m^2^, *P* < 0.001). This difference in eGFR was modulated by donor type and donor age (Figure 3 and Supplementary Figure S1). In the TCMR group, recipients with preDSA had a lower kidney function as compared with recipients without preDSA (eGFR 51 ml/min per 1.73 m^2^ vs. 38 ml/min per 1.73 m^2^ [DD] and 53 ml/min per 1.73 m^2^ vs. 44 ml/min per 1.73 m^2^ [LD], *P* < 0.001).

The contribution of TCMR to long-term graft loss at 10 years was assessed in the subgroup of recipients without preDSA with a follow-up of at least 10 years (*n* = 1086, Table 3). The preDSA recipients were excluded as preDSA have a detrimental influence on long-term graft survival because of the development of chronic-ABMR^16^ which constitutes a major bias in the interpretation of the actual role of TCMR-related graft loss. Overall, rejection-related graft loss at 10 years was 7%. TCMR-related graft loss was 2.9%, ABMR-related graft loss (including mixed type rejection) was 3.3%, and in 0.6% of the cases, the recipient presented with graft loss related to severe medication nonadherence (tacrolimus blood concentration < 3 ug/l) in which case no kidney biopsy was performed.

**Figure 4:**
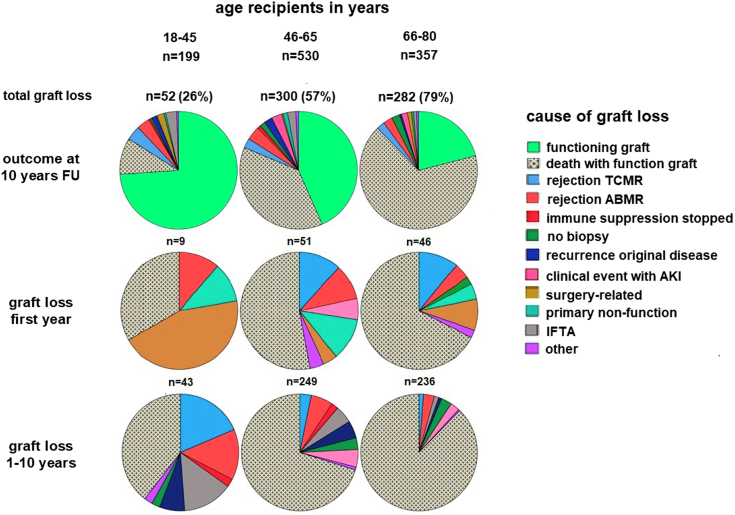
Graft loss at 10-year follow-up for the recipients transplanted without donor-specific anti-HLA antibodies within different recipient age categories. All recipients (*n* = 1086) had at least 10-year follow-up or graft loss occurred within that period. The upper row shows the outcome of all transplantations followed by distribution of cause of graft loss within the first year (middle row) and after the first year (bottom row). Of note, rejections are depicted in blue (T-cell-mediated rejection [TCMR]) or red (antibody-mediated rejection [ABMR]), dark red are cases presenting with sudden graft loss because of severe incompliance. AKI, acute kidney injury, FU, follow-up; IFTA, interstitial fibrosis and tubular atrophy. The numbers of graft loss are shown per distribution circle, and the % of total number of recipients per age category (upper row) are given within parenthesis.

At 10 years follow-up, the overall contribution of TCMR-related graft loss was 4 % in the 18 to 45 yrs recipient group, 2.6% in the 46 to 65 yrs group, and 2.2% in the 66 to 80 yrs group (Figure 4 and Supplementary Table S3). As expected, graft loss because of death was the dominant cause in the older adult recipients.

In conclusion, TCMR is associated with a decreased eGFR at 1 year, but TCMR related graft loss at 10-year post-transplant is relatively low. The presence of DSA at time of transplantation significantly impacts not only the incidence of TCMR but also TCMR-related decrease in eGFR and graft loss at 1 year after transplantation.

### TCMR and Recipient Survival

**Figure 5:**
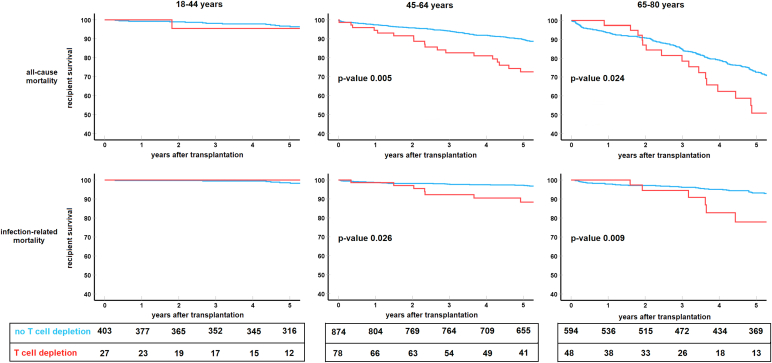
Difference in survival curves for recipients who received T-cell depletion therapy compared with no T-cell depletion therapy. The upper panel shows all-cause mortality Kaplan-Meier survival curves for the different recipient age categories. The lower panel shows the infection-related mortality survival curves. Below the figure, the table shows the number of cases at risk per treatment group and age category.

Cause of death at 5-year follow-up was cardiovascular (25%), malignancy (24%), infection (25%), and other or unknown (26%) (Supplementary Table S4). T-cell depletion was associated with a decreased recipient survival as compared with no T-cell depletion (aHR: 1.69, 95% CI: 1.23–2.34, *P* = 0.001). Further dissection into cause of death showed that T-cell depletion was associated with an increase of infection-related death (aHR: 2.03, 95% CI: 1.16–3.56, *P* = 0.013). Of note, this effect was observed only from 1 to 2 years onwards and only statistically significant in the recipient age groups of 45 years and older (aHR: 2.15, 95% CI:1.16–3.56, *P* = 0.008, Figure 5). This translated into an infection-related death rate of 10% in T-cell depletion versus 4% in those without T-cell depletion (*P* = 0.002) at 5 years follow-up in the recipients aged ≥ 45-years.

## Discussion

This study reports a low rate of long-term TCMR-related graft loss in recipients without preDSA in a large single center cohort of kidney transplant recipients treated with the ELITE immunosuppressive scheme including basiliximab induction and tacrolimus, MMF, or prednisone. The presence of DSA before transplantation increased the risk substantially for all types of TCMR and TCMR-associated graft loss.

To our knowledge, there are no previous data with comparable long-term follow-up published, except for data from a Dutch national registry comprising the period from 1995 to 2005.^2^^,^^19^ The cumulative incidence of TCMR and also TCMR-related episodes leading to graft loss is halved in recipients without preDSA (from 30% to 15% and from 20% to 10%). Compared with this registry the current data also show that the effect of recipient age on TCMR is quenched between the young and older-adult groups (15% difference in cumulative TCMR incidence reduced to less than 10%) and TCMR-related graft loss at 10 years decreased in the last decade from an average of 7% to 4%. In addition, classical immunological risk factors such as retransplantation, extended criteria DDs, and positive PRAs were not associated with TCMR.^20^

The presence of antibodies against highly polymorphic HLA molecules indicates that previous activation of direct and indirect alloreactive T cells may have led to the production of alloreactive memory T cells.^21^ As the frequency of alloreactive memory T cells is a risk factor for TCMR,^22^ an association between preDSA and increased risk for TCMR is plausible. Nevertheless, data on this association have shown conflicting results.^14^, ^15^, ^16^, ^17^ An explanation for this inconsistency is difficult to give as the studied patient groups differ in type of induction (none, anti-CD25, and T-cell depletion) type of maintenance immune suppression (containing ciclosporin, tacrolimus, or no calcineurin inhibitor) and study design (e.g., cohort studies vs. selected cases).

Several findings in our analysis on preDSA in relation to TCMR are worth noting. Only preDSA for HLA class II was associated with the risk for TCMR. This finding agrees with the concept that indirect alloreactive T cells against mismatched HLA class II may be important in facilitating TCMR, but this is currently a hypothetical explanation.^12^^,^^13^ Older age of the donor was positively associated with the frequency of TCMR in the preDSA group. However, this was a modest effect in contrast to the previously described strong negative interaction between donor age and preDSA on ABMR incidence and graft loss. Lastly, the cumulative incidence of TCMR plateaued after 1 to 3 years after transplantation, whereas the cumulative incidence for ABMR did not plateau in recipients with preformed DSA.^10^^,^^14^ Activation-induced death of risky polyfunctional direct alloreactive T cells in the months to years after transplantation may be a mechanism underlying the waning of new cases of TCMR after 3 to 6 months post-transplantation.^23^, ^24^, ^25^

The current results are of clinical interest as they favor the use of basiliximab induction, particularly in the older adults who are more vulnerable for infectious complications.^5^^,^^26^^,^^27^ Our data show excellent long-term outcomes with anti-CD25 induction in combination with tacrolimus and mycophenolate maintenance therapy. The association between T cell depleting antirejection therapy and increased risk for infection-related death warrants restraint in T-cell depletion as induction therapy. As the numbers of older adult transplant recipients are increasing (with > 50% of newly transplanted recipients are > 60 years at our center), basiliximab-tacrolimus-MMF-prednisone may be a preferred initial immunosuppressive regime in these recipients.^28^

The results from this patient cohort show that preformed DSA significantly impact graft survival mediated by early TCMR (this study) as well as ca-ABMR.^10^ Preformed DSA should therefore be avoided, preferably via another LD (e.g., via kidney exchange) or via adjusted DD allocation.^29^^,^^30^ This must be weighed against higher morbidity, mortality, and lower quality of life associated with prolonged dialysis. In addition, removal of potential less harmful DSA by delisting strategies for very highly immunized patients can substantially increase their chance for receiving a donor kidney with acceptable short-term graft survival.^31^^,^^32^

A limitation of the present study is the single center design, but the number of recipients is large with an almost complete database and data of high granularity. Also, no protocol biopsies were taken which may have led to an underestimation of the rejection incidence, and DSA were not measured routinely after transplantation.

In conclusion, TCMR-related graft loss with a basiliximab-tacrolimus-MMF-prednisone based immunosuppressive regime is low. The presence of anti-HLA class II DSA before transplantation substantially increases the incidence of TCMR and TCMR-related graft loss after transplantation.

## Disclosure

All the authors declared no conflicts of interest.

## Supplementary Material

Supplementary file PDF STROBE Checklist. **Figure S1.** Mean estimated glomerular filtration at 1 year after transplantation for different kidney donor types (LD, DBD, and DCD) in relation to age of the donor. **Table S1.** Analysis of risk factors for TCMR in all recipients. **Table S2.** Analysis of risk factors for TCMR in recipients with preDSA excluded. **Table S3.** Cause of graft failure in 10-years follow-up cohort. **Table S4.** Cause of death in T-cell depletion versus no T-cell depletion groups at 5 years follow-up. STROBE Statement.

## Footnotes

Supplementary file (PDF)
**Figure S1.** Mean estimated glomerular filtration at 1 year after transplantation for different kidney donor types (LD, DBD, and DCD) in relation to the age of the donor. **Table S1.** Analysis of risk factors for TCMR in all recipients. **Table S2.** Analysis of risk factors for TCMR in recipients with preDSA excluded. **Table S3.** Cause of graft failure in 10-years follow-up cohort. **Table S4.** Cause of death in T-cell depletion versus no T-cell depletion groups at 5 years follow-up. STROBE Statement.
