# Supplementary material for: Reappraisal of Risk Factors for T-Cell-Mediated Kidney Rejection
Source: Kidney Int Rep. 2026 Apr 13;11(6):106537. doi: 10.1016/j.ekir.2026.106537 (PMC13187526; doi:10.1016/j.ekir.2026.106537)
Supplement: Supplementary file PDF — STROBE Checklist. Figure S1. Mean estimated glomerular filtration at 1 year after transplantation for different kidney donor types (LD, DBD, and DCD) in relation to age of the donor. Table S1. Analysis of risk factors for TCMR in all recipients. Table S2. Analysis of risk factors for TCMR in recipients with preDSA excluded. Table S3. Cause of graft failure in 10-years follow-up cohort. Table S4. Cause of death in T-cell depletion versus no T-cell depletion groups at 5 years follow-up. STROBE Statement. [file mmc1.pdf]

Table S1. Analysis for risk factors TCMR all recipients				
Univariate analysis			Multivariate analysis	
	HR 95% CI	p-value	HR 95% CI	p-value
Age recipient	0.98 (0.98-0.98	0.002	0.98 (0.98-0.99)	<0.001
Age donor	1.01(0.99-1.101	0.08	1.01 (1.00-1.02)	0.013
DD vs LD	1.24 (1.02-1.52)	0.03	1.38 (1.12-1.70)	0.003
Pre-emptive yes/no	1.21 (0.97-1.45)	0.09		
Previous KT	1.21 (1.03-1.41)	0.02		
PRA>5%	1.43 (1.06-1.93)	0.02		
Number of HLA MM	1.09 (1.02-1.16)	0.008	1.09 (1.02-1.16)	0.011
Number of MM on DR	1.11 (0.96-1.29)	0.15		
CIT (hrs)	1.00 (0.99-1.01)	0.23		
preDSA present	2.01 (1.54-2.61)	<0.001	2.37 (1.88-2.99)	<0.001
Male vs Female	1.11 (0.93-1.48)	0.15		

Table S2. Analysis for risk factors TCMR (recipients with preDSA excluded)					
Univariate analysis			Multivariate analysis		
	HR 95% CI	p-value		HR 95% CI	p-value
Age recipient	0.99 (0.98-1.00)	0.04		0.99 (0.98-0.99)	0.004
Age donor	1.01 (0.99-1.01)	0.53			
DD vs LD	1.30 (1.02-1.62)	0.04		1.39 (1.09-1.77)	0.007
Pre-emptive yes/no	1.24 (0.96-1.59)	0.09			
Previous KT	1.12 (0.87-1.44)	0.38			
PRA>5%	1.20 (0.72-1.99)	0.48			
Number of HLA MM	1.1 (1.02-1.19)	0.01		1.24(1.04-1.21)	0.003
Number of MM on DR	1.01 (0.84-1.21)	0.95			
CIT (hrs)	1.00 (0.99-1.01)	0.37			
preDSA present	NA				
Male vs Female	0.83 (0.63-1.07)	0.16			

Table S3. Cause of graft failure in 10-years follow-up cohort				
	All n=1086	18-45 n=199	46-65 n=530	66-80 n=357
**Functioning graft**	452 (42%)	147 (74%)	230 (43%)	75 (21%)
Cause of graft failure				
Death with functioning graft	459 (42.3%)	20 (10.0%)	201 (38%)	238 (66.7%)
TCMR	32 (2.9%)	8 (4.0%)	14 (2.6%)	8 (2.2%)
Mixed rejection	12 (1.1%)	3 (1.5%)	5 (0.9%)	4 (1.1)
ABMR	24 (2.2%)	4 (2.0%)	15 (2.8%)	5 (1.4%)
Immune suppression stopped	6 (0.6%)	1 (0.5%)	5 (0.9%)	0 (0%)
No biopsy	17 (1.6%)	1 (0.5%)	8 (1.5%)	8 (2.2%)
Recurrence original disease	17(1.6%)	3 (1.5%)	12 (2.3%)	2 (0.6%)
Clinical event with AKI	21 (1.9%)	0 (0%)	15 (2.8%)	6 (1.7%)
Surgery-related	10 (0.9%)	4 (2%)	2 (0.4%)	4 (1.1%)
Primary non-function	10 (0.9%)	1 (0.5%)	7 (1.3%)	2 (0.6%)
IFTA	21 (1.9%)	6 (3%)	12 (2.3%)	3 (0.8%)
other	7 (0.6%)	1 (0.5%)	4 (0.8%)	2 (0.6%)

Table S4. Cause of death in T cell depletion vs. no T cell depletion groups at 5 years follow up.				
	No T cell depletion N=1871		T cell depletion given N=153	p-value
Total deceased (% of total recipients)	247 (13%)		32 (22%)	P=0.003
Cardiovascular cause<br>% of total recipients	62 (25%)<br>3.3%		13 (38%)<br>8.5%	P=0.003
Malignancy<br>% of total recipients	59 (24%)<br>3.2%		5 (15%)<br>3.2 %	
Infection<br>% of total recipients	61 (25%)<br>3.2%		13 (38%)<br>8.5%	P=0.003
Other<br>% of total recipients	17 (7%)<br>0.9%		2 (6%)<br>1.3%	

Unknown<br>% of total recipients	48 (19%)<br>2.6%		1 (2.9%)<br>0.6%	

Figure S1

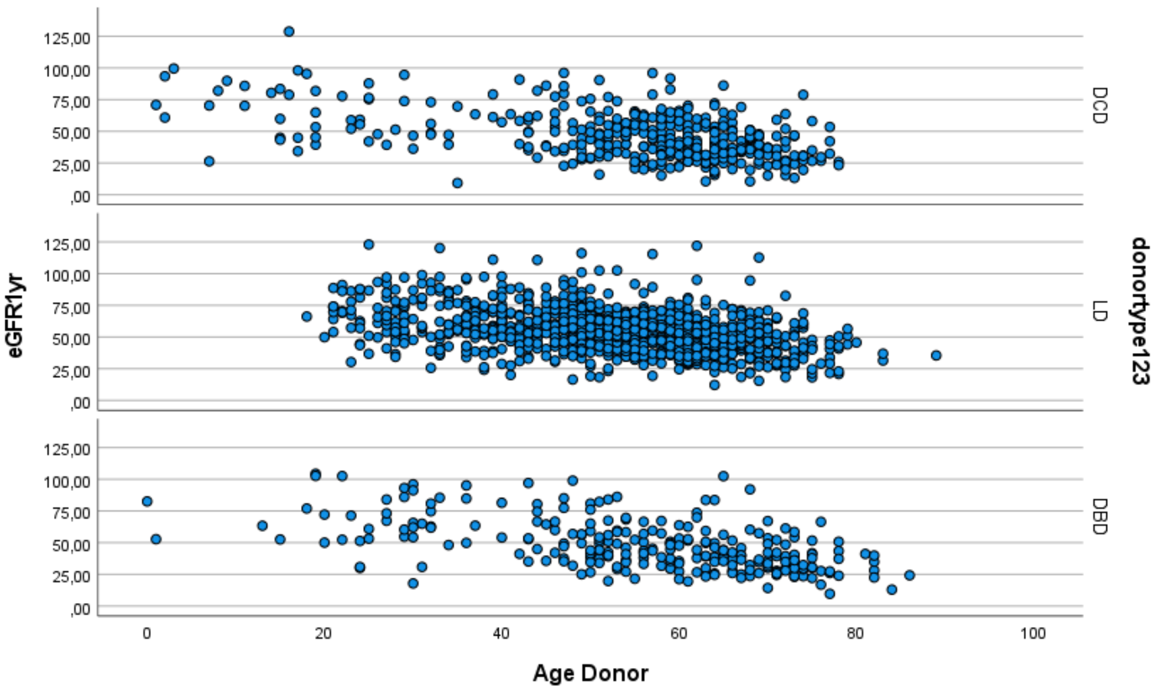

125,00
100,00
75,00
50,00
25,00
,00
DCD
LD
DBD
eGFR1yr
donortype123
0
20
40
60
80
100
Age Donor

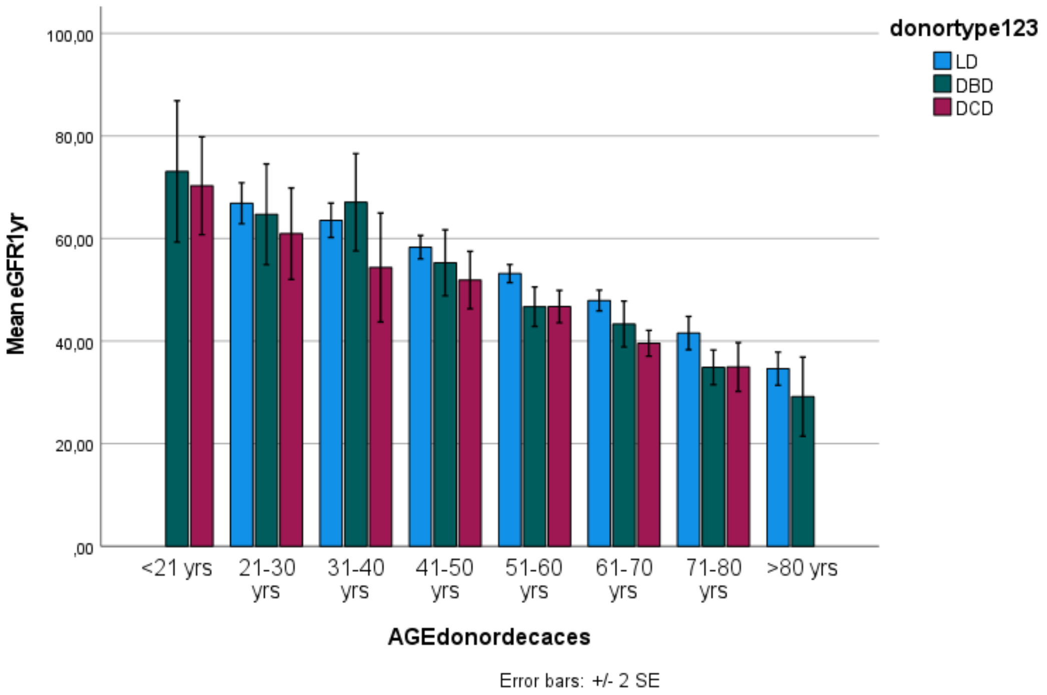

Mean estimated glomerular filtration (eGFR) at 1 year after transplantation for different kidney donor types (living (LD) donor, donation after brain death (DBD), donation after cardiac death (DCD)) in relation to age of the donor.

STROBE Statement—checklist of items that should be included in reports of observational studies

	Item No.	Recommendation	Page No.	Relevant text from manuscript
**Title and abstract**	1	(*a*) Indicate the study's design with a commonly used term in the title or the abstract	2	A single center cohort of 2024 kidney transplant recipients between 2010-2020 with follow up until June 2025 was analyzed retrospectively
		(*b*) Provide in the abstract an informative and balanced summary of what was done and what was found	2	An immune suppressive regime of anti-CD25 induction and tacrolimus/mycophenolate mofetil/prednisone maintenance is associated with a low risk of TCMR-related graft loss. The presence of DSA against HLA class II before transplantation constitutes a major risk factor for TCMR-mediated graft loss.
**Introduction**				

Background/rationale	2	Explain the scientific background and rationale for the investigation being reported	4	.... lacking of long term follow-up data on efficacy and adverse effects of the ELITE tacrolimus-based scheme without selection of recipients for T cell depletion or basiliximab.<br>....the association between preDSA and incidence of TCMR has has shown contradictory results
Objectives	3	State specific objectives, including any prespecified hypotheses	4	We investigated the cumulative risk for TCMR and associated short and long term graft loss, and reevaluate risk factors for TCMR taking preDSA into account, in a cohort from 2010 till 2020 with granular data
**Methods**				
Study design	4	Present key elements of study design early in the paper	5	This study included all 2,124 consecutive kidney transplantations performed between

January 2010 and December 2020 at the Erasmus Medical Center in the Netherlands. The last follow-up date for data analysis was June 2025. Recipients were seen at least once a year at the out-patient clinic, and clinical data were registered in a national database (Netherlands Organ Transplant Registry). All transplantations across the ABO blood group barrier (n = 88) or a positive complement-dependent cytotoxicity cross-match at time of transplantation (n = 12) were excluded from analysis. The clinical characteristics of the included 2024 patients are shown in Table 1.

Setting	5	Describe the setting, locations, and relevant dates, including periods of recruitment, exposure, follow-up, and data collection	5	consecutive kidney transplantations performed between January 2010 and December 2020 at the Erasmus Medical Center in the Netherlands. The last follow-up date for data analysis was June 2025. Recipients were seen at least once a year at the out-patient clinic, and clinical data were registered in a national database (Netherlands Organ Transplant Registry).
Participants	6	(*a*) *Cohort study*—Give the eligibility criteria, and the sources and methods of selection of participants. Describe methods of follow-up<br>*Case-control study*—Give the eligibility criteria, and the sources and methods of case ascertainment and control selection. Give the rationale for the choice of cases and controls<br>*Cross-sectional study*—Give the eligibility criteria, and the sources and methods of selection of participants	5	All transplantations across the ABO blood group barrier (n = 88) or a positive complement-dependent cytotoxicity cross-match at time of transplantation (n = 12) were excluded from analysis.

		(*b*) *Cohort study*—For matched studies, give matching criteria and number of exposed and unexposed<br><br>*Case-control study*—For matched studies, give matching criteria and the number of controls per case		
Variables	7	Clearly define all outcomes, exposures, predictors, potential confounders, and effect modifiers. Give diagnostic criteria, if applicable	6	All renal biopsies were for cause and were performed in case of progressive loss of graft function. The initial biopsy reviews were rescored following the 2018 Banff Reference Guide[18]. T cell-mediated rejection was treated with methylprednisolone 1000 mg intravenously for 3 days in case of borderline (bTCMR) or TCMR 1 (tubulo-interstitial rejection). TCMR 2-3 (vascular rejection) was treated with T cell depletion therapy using rabbit anti-thymocyte immunoglobulin between 2010-2015 or

Alemtuzumab from 2015 to 2020.

Outcomes and variables

For data analysis, histology was further categorized as previously published [19]: rejection, recurrence of primary kidney disease, diagnosis of de novo kidney disease, and interstitial fibrosis with tubulus atrophy (IFTA). In case of graft failure, the diagnosis of for cause kidney biopsies was used to categorize the type of graft failure if no other clinical event could explain the loss of kidney function.

The other graft loss categories were a clinical event leading to irreversible graft failure

			(e.g., circulatory shock, pyelonephritis, graft thrombosis) and "unknown" if a clinical diagnosis for allograft failure could not be established and no biopsy was performed (1.2% of all cases of graft loss other than death). Primary non-function is the category of grafts that never functioned after transplantation with no other diagnosis than acute tubular necrosis (ATN) as shown by kidney biopsy. Anti-HLA donor-specific antibodies were measured in this study as previously reported [16]. The percentage of panel reactive antibodies (PRA) at time of transplantation, as determined by complement-dependent

cytotoxicity assay, was considered positive when >5%.

The relative contribution of TCMR-related graft loss was assessed at 1 year after transplantation and for the group of recipients with a follow-up of at least 10 year and no preDSA (Table 2). The latter was group was selected in order to account for death with a functioning graft as competitive risk factor within different recipient age strata.

Cause of death was categorized as infection-related, cardiovascular, malignancy, other and unknown.

Data sources/ measurement	8*	For each variable of interest, give sources of data and details of methods of assessment (measurement). Describe comparability of assessment methods if there is more than one group	6	For data analysis, histology was further categorized as previously published [19]: rejection, recurrence of primary kidney disease, diagnosis of de novo kidney disease, and interstitial fibrosis with tubulus atrophy (IFTA). In case of graft failure, the diagnosis of for cause kidney biopsies was used to categorize the type of graft failure if no other clinical event could explain the loss of kidney function.<br><br>The other graft loss categories were a clinical event leading to irreversible graft failure (e.g., circulatory shock, pyelonephritis, graft thrombosis) and "unknown" if a clinical diagnosis for allograft failure could not be

				established and no biopsy was performed (1.2% of all cases of graft loss other than death). Primary non-function is the category of grafts that never functioned after transplantation with no other diagnosis than acute tubular necrosis (ATN) as shown by kidney biopsy. Anti-HLA donor-specific antibodies were measured in this study as previously reported [16]. The percentage of panel reactive antibodies (PRA) at time of transplantation, as determined by complement-dependent cytotoxicity assay, was considered positive when >5%.
Bias	9	Describe any efforts to address potential sources of bias	6	"unknown" if a clinical diagnosis for allograft failure could not be

				established and no biopsy was performed (1.2% of all cases of graft loss other than death).
Study size	10	Explain how the study size was arrived at	5	consecutive kidney transplantations performed between January 2010 and December 2020 at the Erasmus Medical Center in the Netherlands.

Continued on next page

Quantitative variables	11	Explain how quantitative variables were handled in the analyses. If applicable, describe which groupings were chosen and why	5	The relative contribution of TCMR-related graft loss was assessed at 1 year after transplantation and for the group of recipients with a follow-up of at least 10 year and no preDSA (Table 2). The latter group was selected in order to account for death with a functioning graft as competitive risk factor within different recipient age strata.
Statistical methods	12	(*a*) Describe all statistical methods, including those used to control for confounding	6	Differences in patient, donor, and transplant characteristics were assessed by the Fisher's exact test for categorical variables and Mann–Whitney U test for continuous variables. All p-values were two-tailed. The

cumulative incidence of TCMR and death-censored graft loss was assessed by Kaplan–Meier survival analysis with log-rank statistics for difference between strata. Univariate Cox proportional hazards analysis was used to identify clinical and demographic variables associated with rejection and graft survival. Variables considered for analysis were as follows: donor age, age of recipient, re-transplantation, pre-emptive transplantation, positive PRA, pretransplant DSA, number of HLA mismatches on A, B and DR, type of kidney donor (LD, DCD, DBD), male/female, and cold ischemia time. Variables with a p-value of <0.1 in a

			univariate analysis were subsequently used in the multivariate Cox proportional hazard analysis with stepwise forward regression to calculate adjusted hazard ratios for the outcome (e.g., TCMR or graft failure).
	(*b*) Describe any methods used to examine subgroups and interactions	6	Interaction terms that met statistical significance ($p < 0.05$) were included in the multivariate model
	(*c*) Explain how missing data were addressed	5	Recipients were seen at least once a year at the out-patient clinic, and clinical data were registered in a national database (Netherlands Organ Transplant Registry) complemented with data from the local registry. The final database for analysis was >98% complete for all variables.

		(*d*) *Cohort study*—If applicable, explain how loss to follow-up was addressed<br><br>*Case-control study*—If applicable, explain how matching of cases and controls was addressed<br><br>*Cross-sectional study*—If applicable, describe analytical methods taking account of sampling strategy	7	Thirty-eight recipients (1.9%) were lost to follow-up at a median time of 2 years after transplantation.
		(e) Describe any sensitivity analyses		n.a.
**Results**				
Participants	13*	(a) Report numbers of individuals at each stage of study—eg numbers potentially eligible, examined for eligibility, confirmed eligible, included in the study, completing follow-up, and analysed		Numbers of recipients at risk given for every outcoe and within groups are given in the legends of the figures
		(b) Give reasons for non-participation at each stage		n.a.
		(c) Consider use of a flow diagram		n.a.
Descriptive data	14*	(a) Give characteristics of study participants (eg demographic, clinical, social) and information on exposures and potential confounders		Two tables with relevant data
		(b) Indicate number of participants with missing data for each variable of interest		Given in tables and figures
		(c) *Cohort study*—Summarise follow-up time (eg, average and total amount)		Table 1
Outcome data	15*	*Cohort study*—Report numbers of outcome events or summary measures over time		Given in Tables and figures
		Case-control study—Report numbers in each exposure category, or summary measures of exposure		
		Cross-sectional study—Report numbers of outcome events or summary measures		

Main results	16	(*a*) Give unadjusted estimates and, if applicable, confounder-adjusted estimates and their precision (eg, 95% confidence interval). Make clear which confounders were adjusted for and why they were included	7-9	Givin within text
		(*b*) Report category boundaries when continuous variables were categorized		done in figures
		(*c*) If relevant, consider translating estimates of relative risk into absolute risk for a meaningful time period	9	This translated into an infection-related death rate at 5 years follow-up in the ≥45 year recipients of 10.3% in T cell depletion vs. 3.8% without T cell depletion (p=0.002).

Continued on next page

Other analyses	17	Report other analyses done—eg analyses of subgroups and interactions, and sensitivity analyses		Reported in supplemental tables
**Discussion**				
Key results	18	Summarise key results with reference to study objectives	10	This study reports a low rate of long term TCMR-related graft loss, unless donor-specific HLA-antibodies were present before transplantation in a large single center cohort of kidney transplant recipients treated with the ELITE immunosuppressive scheme including basiliximab induction and tacrolimus/MMF/prednisone. The presence of DSA before transplantation increased the risk for all types of TCMR and associated graft loss by threefold.
Limitations	19	Discuss limitations of the study, taking into account sources of potential bias or imprecision. Discuss both direction and magnitude of any potential bias	11	A limitation of the present study is the single center design, but the number of recipients is large with an almost complete database

				and data of high granularity were evaluated.
Interpretation	20	Give a cautious overall interpretation of results considering objectives, limitations, multiplicity of analyses, results from similar studies, and other relevant evidence	10	There are no previous data with comparable long term follow-up published, except for data from a Dutch national registry comprising the period 1995-2005 [2, 19]. The cumulative incidence of TCMR but also TCMR-related graft loss episodes leading to graft loss is almost halved (from 30% to 17% and from 20% to 10%). Compared to this registry the current data show that the effect of recipient age on TCMR quenched between the young and elderly groups (15% difference in cumulative TCMR incidence to less than 10%) and TCMR-related graft loss at 10 years decreased in the last decade from an average of 7% to 2%.

Generalisability	21	Discuss the generalisability (external validity) of the study results		
**Other information**				
Funding	22	Give the source of funding and the role of the funders for the present study and, if applicable, for the original study on which the present article is based	12	The author(s) declare that no financial support was received for the research, authorship, and/or publication of this article.

*Give information separately for cases and controls in case-control studies and, if applicable, for exposed and unexposed groups in cohort and cross-sectional studies.
